# The Roles of Dimensionality, Canopies and Complexity in Ecosystem Monitoring

**DOI:** 10.1371/journal.pone.0027307

**Published:** 2011-11-03

**Authors:** Christopher H. R. Goatley, David R. Bellwood

**Affiliations:** Australian Research Council Centre of Excellence for Coral Reef Studies, and School of Marine and Tropical Biology, James Cook University, Townsville, Queensland, Australia; Institute of Marine Research, Norway

## Abstract

Canopies are common among autotrophs, increasing their access to light and thereby increasing competitive abilities. If viewed from above canopies may conceal objects beneath them creating a ‘canopy effect’. Due to complexities in collecting 3-dimensional data, most ecosystem monitoring programmes reduce dimensionality when sampling, resorting to planar views. The resultant ‘canopy effects’ may bias data interpretation, particularly following disturbances. Canopy effects are especially relevant on coral reefs where coral cover is often used to evaluate and communicate ecosystem health. We show that canopies hide benthic components including massive corals and algal turfs, and as planar views are almost ubiquitously used to monitor disturbances, the loss of vulnerable canopy-forming corals may bias findings by presenting pre-existing benthic components as an altered system. Our reliance on planar views in monitoring ecosystems, especially coral cover on reefs, needs to be reassessed if we are to better understand the ecological consequences of ever more frequent disturbances.

## Introduction

Worldwide, the increasing frequency and severity of ecosystem disturbances associated with changing climatic conditions and direct anthropogenic activities has increased the need for ecosystem monitoring. Effective monitoring programmes aim to detect disturbances in time to mitigate their impacts [1], [2]. To be effective, these monitoring programmes must be fast and cost-effective, as this facilitates repeated observations, a vital feature to detect changes in ecosystems. While a plethora of methods exist to monitor habitats across various ecosystems, one feature is almost universal among direct monitoring regimes regardless of ecosystem: the use of horizontal planar views in sampling. This standardises monitoring and provides rapid assessments of abundance or cover of organisms [3]–[5]. A potential concern arises, however, as ecosystems are inherently 3-dimensional. By reducing dimensionality in monitoring we simplify data collection and analysis, but at what cost to the quality of data?

Obviously, recording 3-dimensional data from ecosystems is highly complex and increases the amount of time needed to sample. Reducing dimensionality in sampling is therefore justified if the increased speed and reduced cost improves the spatial and/or temporal resolution of the data being collected. Of the 3 dimensions, however, it has almost always been the vertical component that is discarded first, leaving the horizontal axes. The implications of this reliance on linear or planar views could affect the data collected in ecosystem monitoring, particularly with regards to the detection of disturbances in multi-layered ecosystems. Furthermore, as these methods have often been extended for use in more detailed ecological studies, biases associated with reducing dimensionality could undermine our understanding of key ecological processes in complex ecosystems.

Alongside tropical rainforests, coral reefs are one of the most biodiverse ecosystems on the planet [6]. Furthermore, coral reefs are arguably the most threatened high biodiversity ecosystem. They are highly sensitive to physical and environmental perturbations [7]–[9]. As such, many monitoring programmes have been implemented, some over several decades [4], [10]. The methods used, therefore, are relatively well developed (e.g. [4]), and numerous detailed ecological studies have used similar methods in their data collection [11]–[13]. Almost all of these methods, however, are based on horizontal linear or planar views, and the most commonly reported metric of reef health is coral cover [10], [14]. At present, the limitations of planar or linear views in coral reef studies are poorly understood. Given the fine scale, 3-dimensional structural complexity of corals, coral reefs represent an ideal model ecosystem to consider the effects of reduced dimensionality in sampling methods, and as such they are the primary focus of this study.

### Potential problems with planar views

The use of planar views while studying and monitoring ecosystems has the potential to create problems falling into several general categories. The first problem is the development of a ‘methodological inertia’. The widespread and historical use of planar sampling techniques (e.g. [15]–[17]), like blinders on a horse, set a course for future studies to use similar methods. The simplicity of data collection and interpretation from planar views has exacerbated this problem. Such simple methods can easily be transposed between ecosystems [18]–[20] and in some cases the ecological or structural differences between these ecosystems may have been overlooked. Modern technologies such as still and video cameras, and even aerial and satellite surveys (e.g. [5], [21]–[22]) have increased the amount of field data that can be collected, however, these images are almost exclusively planar. While the areas sampled may be bigger, the methods are essentially the same.

A second problem is that collecting 3-dimensional data is challenging. This is particularly the case where rapid and repeatable observations are needed, as per most monitoring programmes. Manual collection of 3 dimensional data is impractical. The use of stereoscopic images [23]–[25] and other recent developments allow some level of 3-dimensional sampling [26], [27] but most of these technologies are not widely used in large-scale studies due to the complexity of data collection and analysis. As such, 3-dimensional ecosystems are most commonly monitored in 1- or 2-dimensions.

Potentially the biggest problem with planar views is that the vertical component of ecosystems is often lost during periods of ecological change. By overlooking the vertical relief of ecosystems no data on structural complexity can be derived. However, structural complexity is of considerable importance in facilitating the development of high biodiversity and resilience. A highly complex ecosystem provides cover for prey [28], [29], allows predators to find effective ambush sites [30], and increases environmental niches, reducing the severity of physical stresses (e.g. [31], [32]). Furthermore, for coral reefs and rainforests alike, one of the most serious post-disturbance effects is the loss of structural complexity associated with the collapse of the biogenic habitat [33]–[35]. Standard planar or linear views of ecosystems do not provide information on this property, thus their utility in documenting and understanding the effects of disturbance is limited.

### The Canopy Effect

Autotrophs' need for light has led to the recurring development of large canopy shaped growth forms to both overtop competitors and increase the surface area exposed to light. These structures almost invariably become biogenic habitats themselves. Using planar or linear views to sample any of these habitats can cause a bias in sampling, in terms of a ‘canopy effect’. Perhaps the best conceptual example of this would be from tropical rainforests. While planar views from aerial or satellite imaging provide useful information on the areas covered by these ecosystems, little data is provided on any organisms below the upper canopy as they are hidden beneath it. This effect is not, however, constrained to rainforests. In fact, if any observed substrate has 3-dimensional structure it is likely that the upper layers will obscure those below them. This effect is more acute if the upper layers occupy more horizontal space at elevation than they do beneath (i.e. form a canopy). Canopy effects, therefore, can potentially occur at any scale from microscopic samples in a Petri dish to satellite imaging of entire forest ecosystems.

In comparison to rainforests, coral reefs could be especially prone to canopy effects. Their relatively small vertical elevations mean that, unlike rainforests, we cannot easily observe the ecosystem from within, and as such the layered structure is easier to overlook. Furthermore, pronounced canopies, and the logistical challenges associated with accessing them accentuate this problem. As such, in this study we use data collected from coral reefs to explore and quantify the canopy effect with an emphasis on potential implications in monitoring disturbances.

### Coral reefs and the ‘canopy effect’

While it might be presumed that coral reefs are coral dominated, this is rarely the case [14], [36]. Coral reefs are, of course, defined by the presence of scleractinian corals, but at present they rarely represent the primary benthic cover of these habitats. A meta-analysis revealed that the mean scleractinian coral cover on coral reefs from 88 locations worldwide was 25.9±1.5% (mean ± S.E.; data from [10], [37]). As the methods used to collect these data are entirely based upon linear or planar views, from reefs likely to be dominated by canopy-forming species, but without considering a canopy effect, the actual benthic cover of corals is likely to be much lower. Bare space is, however, almost non-existent on most reefs [38]–[40] and, as such, the remaining three quarters of the benthos of coral reefs is relatively poorly understood (cf. [14], [36], [41]).

Although corals are arguably the most important organisms on coral reefs, especially in terms of growth and accretion, numerous other benthic organisms play measurable ecological roles on reefs [42]–[44]. Calcareous and filamentous algal turfs, for example, are ubiquitous components of coral reef flora, potentially occupying more of the reef than corals [14], [38], [40]. Calcareous turfs aid reef calcification [45]–[47], while filamentous turfs create an epilithic algal matrix (EAM) containing sediments, detritus and infaunal organisms [48], which can reduce the settlement and survivorship of coral larvae [38], [49] and reduce the palatability of turfs to herbivores [50]. The effects of algal turfs are becoming more apparent, yet their actual benthic cover on coral reefs is essentially unknown; we will provide a preliminary estimation of the benthic cover of algal turfs on coral reefs.

Coral reefs are not uniform. Scleractinian corals are phylogenetically and morphologically diverse and different growth forms often dominate different habitats. Exposed reef crests are often dominated by fast growing branching and tabulate corals (e.g. *Acropora* spp.), which form extensive canopies. More sheltered reefs can be dominated by slow growing massive colonies (e.g. *Porites* spp.), which, with mound shaped morphologies offer little canopy cover. If canopy effects have measurable consequences they should be more pronounced on reefs dominated by branching and tabulate corals than those dominated by massive corals.

## Methods

**Figure 1:**
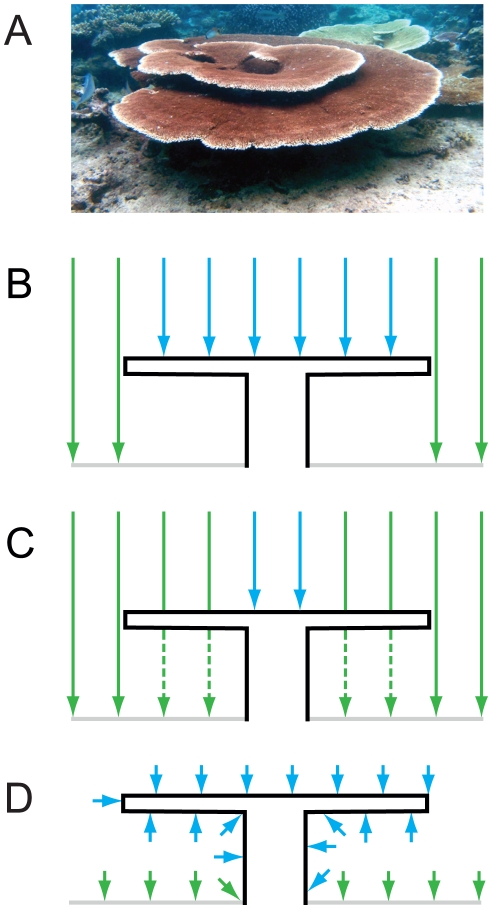
Sampling methods for estimating benthic cover of canopy-forming corals. (**A**) A typical colony of *Acropora hyacinthus* on reefs at Lizard Island. (**B**), A schematic figure demonstrating intercepts (vertical lines; blue  =  coral, green `other benthos) on a planar transect, coral cover  = 60%. (**C**), A schematic figure demonstrating benthic transects, here the dotted vertical lines indicate measurements made beneath the canopy; here coral cover  = 20%. (**D**), A schematic figure demonstrating the chain transects, where lines indicate that measurements were made at set distances along a line that conforms to the outline of the coral; here coral cover  = 68%.

We applied three linear transects along the same course: firstly, a planar point intercept transect (hereafter: planar transect) recording the apparent benthic cover visible from above (as would be seen in a photographic image of the reef) at set intervals, secondly a benthic point intercept transect (hereafter: benthic transect), identical to the planar transect except that the actual benthic cover of the consolidated reef matrix (i.e. the benthic cover beneath any overhangs or canopies) was recorded. A comparison of the first two methods highlights the magnitude any canopy effect on that reef. Finally a chain intercept transect (hereafter: chain transect), which conforms to the benthos below the initial tapes provides both a measure of the vertical relief (rugosity; see [51]–[52]) and the benthic cover across both horizontal and vertical axes (Figure 1). These methods were employed on healthy *Acropora*- and *Porites*-dominated reefs around Lizard Island on the northern Great Barrier Reef to provide a basis on which to illustrate the canopy effect.

## Results

### The ‘canopy effect’ hides algae on coral reefs

**Figure 2:**
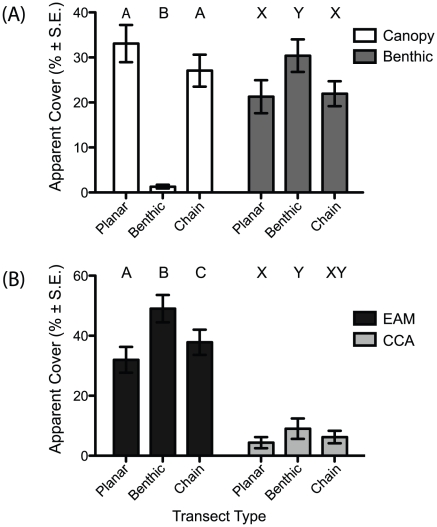
Benthic cover of algae and corals using three transect methods on an *Acropora*-dominated reef. **(A)** The estimated cover of corals on an *Acropora* dominated reef using the three different transect types; canopy forming taxa in white and benthic cover species in dark grey. **(B)** The estimated cover of algae on the same transects; black bars represent cover of epilithic algal matrix (EAM) and light grey, crustose coralline algae (CCA). A, B, C and X, Y, Z denote statistically different groupings (Repeated Measures ANOVA with Tukey Kramer post-hoc test, α  = 0.05).

Regardless of reef type or site, several patterns became apparent when the sampling methods were compared. Most striking is that, using standard planar transects, all reefs showed coral as the dominant benthic component (25.9±2.1% to 54.4±4.9%; mean ± S.E.) and algal turf (EAM) cover as the second most abundant (21.4±1.7% to 34.9±2.7%). Yet when considering cover beneath the canopy (using the benthic transects) a marked change can be seen on the *Acropora-*dominated reefs. The coral cover dropped by almost half from 53.5±2.6% planar cover to 27.7±2.3% in benthic cover. Concurrently, the cover of turf algae (EAM) increased by more than two thirds (from 26.7±2.6% to 44.7±2.6%) becoming the dominant benthic cover on the reefs (Figure 2). The canopy effect essentially hides this portion of the benthos from planar views, and as such, it is overlooked in standard monitoring practices.

Surprisingly, the chain transects, which provide an indication of vertical relief and the surface area of each benthic component, did not provide greatly differing results of coral cover from the planar point intercept transects (coral cover from 29.1±1.5% to 49.0±3.9%). The ‘double counting’ of canopies increases the proportion of coral cover, much as the canopy effect artificially increases the proportion of the benthos seen to be canopies. While coral cover showed similar results between methods, some benthic components, such as calcareous algae, which tended to grow on vertical surfaces, showed significant differences between the benthic and chain transects (on average, chain transects provided estimates of calcareous algal cover 96.4±21.0% higher than planar transects; Figure S1).

## Discussion

### Calcification and the canopy effect

Different colony morphologies of corals provide very different functions on reefs. The planar methods used in most monitoring programmes provide only a limited ability to distinguish these functions. For example, the fast growth and high contribution to coral cover of canopy-forming corals are not necessarily related to the amount of carbonate they deposit on consolidated reef structures. Most canopy-forming species have low density, perforate branches that are easily removed by corallivores [53]–[55] and hydrodynamic forces [56], with much of the carbonate being lost from the reef matrix. As such, with the possible exception of the dense stems of these colonies [57], canopy-forming corals provide a relatively small contribution to reef calcification yet they conceal massive corals and crustose coralline algae that deposit carbonate directly onto the reef matrix. High coral cover, therefore, does not mean high accretion rates [14]. Furthermore, additions to reef carbonates as a result of rubble formation by high cover of canopy-forming corals (e.g. [58]) could be ecologically deleterious as the mobile substrate produced in this process can hinder recruitment of corals and subsequent reef recovery [12], [59], [60].

### Disturbances and the Canopy Effect

The canopy effect, as demonstrated above, influences what we monitor on coral reefs. The overlooked understory has a markedly different composition to the canopy. If reefs were stable, this would result solely in a gap in our ecological understanding of reefs. However, reefs and other ecosystems are most often monitored to observe changes in ecosystem composition brought about by disturbances. The canopy effect is likely to play an important role in our ability to monitor the effects of, and recovery from these disturbances. This may be particularly important if there are differences in the way in which canopy and non-canopy components of the reef respond to disturbances.

### Differential susceptibility and disturbances

Differences between coral colonies are more than morphological. Branching and tabulate taxa such as acroporids, which form the majority of the canopies studied herein, are also the most susceptible to environmental and physical disturbances [61], [62]. They are among the first corals to bleach under environmental stresses [11], often suffering considerable mortality [63]. Physical disturbances also readily dislodge these colonies from the benthos [56] and many coral predators preferentially target these taxa [53], [64], [65]. Massive colonies such as *Porites*, in contrast, are more tolerant to disturbances, being more hydrodynamically stable [56]. They also bleach less readily [63], and are a less favoured prey of corallivores (e.g. [53]).

In almost all cases, canopy-forming corals are most severely affected following disturbances on coral reefs [56], [63] and if the colony's skeleton is left intact following the disturbance they are quickly removed from the reef by biological and physical erosion [9], [66], [67]. The increased susceptibility of coral canopies to disturbances may, therefore, create a bias when monitoring the effects of disturbance on coral reefs.

### Canopy loss and coral cover

**Figure 3:**
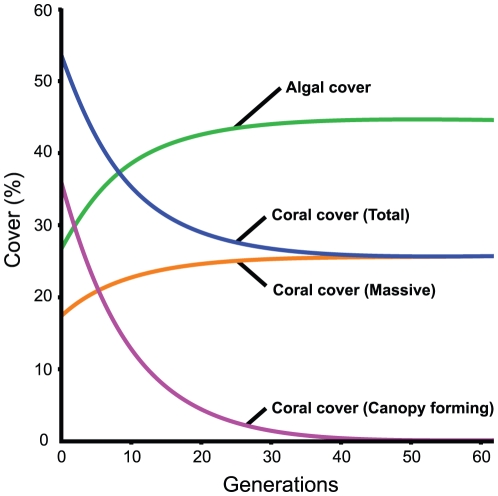
Conceptual model demonstrating the effect of canopy removal on an *Acropora*-dominated reef. Note the apparent increase in both algae (EAM) and massive corals simply as a result of the loss of the canopy. Start points are based on data collected using planar transects. Canopy-forming cover is reduced by 10% each generation. End points are based on data collected using benthic transects (with no remaining canopy).

A simple conceptual model was created using the transect data to assess the magnitude of the canopy effect on *Acropora-*dominated reefs. The high susceptibility of canopy-forming corals to disturbances often results in extensive loss of these corals from reefs [13], [60], [63]. The model replicates this effect. The start point was set as the mean planar cover of algae and corals found on the two *Acropora*-dominated reefs in this study, using the planar transects. The cover of canopy-forming corals is then reduced by 10% each generation for 62 generations (until canopy cover <0.05%) to simulate a disturbance. Using the inverse of canopy cover and the data from the benthic transects, the apparent change in benthic cover is simulated. The cover of concealed benthic components (end point of the model) was based on the results of the benthic transects (Figure 3). A similar model was created using data from the *Porites*-dominated reefs.

As would be expected, on *Porites*-dominated reefs a total loss of canopy-forming corals causes relatively little change (Figure S2). Overtopping is rare on these reefs, as the colonies of massive corals are too large. The only effects seen with the loss of canopy-forming corals is an equivalent reduction of overall coral cover and similar apparent increase of EAM cover.

In contrast, the effect on *Acropora*-dominated reefs is much greater (Figure 3). As canopy-forming corals are competitively dominant on these reefs, predominantly by overtopping other corals, there is a pronounced canopy effect. The loss of canopy-forming species most obviously reduces overall coral cover. However, an apparent 8.2% increase in the cover of massive corals was recorded. This increase indicates that almost a quarter of the space beneath coral canopies was occupied by massive coral colonies. The reported increase in massive coral cover is, therefore, an artefact of using sampling methods based on horizontal planar views in an ecosystem with pronounced canopy effects. However, there are further implications. The recorded loss of coral cover associated with the loss of the canopy is effectively cushioned as the massive colonies are revealed. As coral cover is among the most commonly reported metrics of reef health, the impacts of disturbances may, therefore, be under-reported using planar monitoring methods.

That coral cover provides a somewhat unrealistic measure of the effects of disturbances is not necessarily always this misleading, as most monitoring programmes record far more data than just coral cover. Although there is a clear trade off between precision and spatio-temporal resolution almost all coral monitoring programmes include at least some form of categorisation of the corals observed. Nevertheless, coral cover remains the most widespread metric for communicating reef health. Unfortunately, changing coral cover does not reveal the underlying mechanisms (e.g. recruitment limitation and/or adult mortality), nor can it identify the causes of disturbances. Furthermore, coral cover probably underestimates the magnitude of coral loss and the prevalence of other benthic components. There is the potential, therefore, that researchers may be led astray by just using planar data on reefs and in other 3-dimensional systems.

### Canopy loss and algal cover

The removal of a canopy reveals the other benthic components, which on many reefs, including those selected for this case study, are primarily algal turfs (EAMs). Simulated removal of the canopy from *Acropora*-dominated reefs resulted in a 67% increase in cover of EAMs (Figure 3). While phase-shifts to algal dominated states are among the most reported effects following disturbances on coral reefs [47], [68], [69] our results suggest that in some cases, apparent shifts could simply be due to the canopy effect, with the removal of the coral canopy unveiling a pre-existing algal-dominated state (e.g. [14], [70]. No further ecological succession to an alternate stable ecosystem would be necessary to create an apparent phase-shift to EAMs (turfs) following the loss of these canopy-forming species. Furthermore, exposure of this EAM could possibly trigger an expansion of macroalgae (see [68], [69], [71], [72]).

As discussed above, high benthic cover of EAMs has both positive and negative implications for coral reefs. Algal turfs in the EAM provide settlement cues for a variety of organisms including corals [73], and a habitat for infaunal detritivores, which provide a trophic pathway to recycle energy from the detritus (see [41], [74]). Furthermore, well-grazed EAMs indicate high levels of herbivory by reef fish or invertebrates (see [72]). However, it is likely that a disturbance that removes a high percentage of branching corals is likely to involve multiple synergistic stressors (e.g. [60], [75]). Stressors that affect coral cover may have markedly different effects on the other benthic ecosystem components. The EAM, for example, is considerably more resilient than corals to most environmental perturbations [49], [76], [77]. Indeed, increased temperatures, sediment and nutrients; all stressors to corals, can be beneficial to the algal component of the EAM [78]. Furthermore EAMs are tolerant of high sediment loads [38], [79] and their complex structure slows surface flow and increases sediment deposition [80], [81]. Sediment loaded turfs inhibit the settlement of coral larvae [38] and deter herbivores on coral reefs [50]. The effects of multiple synergistic stressors might, therefore, increase the damage done to reefs not just in terms of the amount of coral damaged but in reducing the recovery potential of reefs, even from an apparently pre-existing algal dominated state.

### Which sampling method is best?

Each of the methods used in this study have benefits and drawbacks, which are important to consider before designing any study. Planar transects, for example, provide a focussed assessment of more susceptible, canopy-forming species (e.g. *Acropora* sp.). As such, planar transects allow rapid detection of disturbances, such as temperature anomalies, which will provoke stress responses (e.g. bleaching) or localised mortality of canopy-forming species [11], [61], [62]. Furthermore, planar transects are quick and easy to complete, allowing high replication at any site. Underwater video and photographs can also be used to collect planar data even by inexperienced observers, with minimal training. However, the present study has revealed several drawbacks with this method. Apparent coral loss following major disturbances might be cushioned as less susceptible benthic corals are revealed. Also, by revealing pre-existing benthic communities, canopy loss could lead to misleading reports of phase-shifts with apparent increases in the cover of algae and massive corals (the changes are relative not actual). This method is excellent for studies of tabulate corals but has serious limitations for other benthic components.

Benthic transects provide useful information on the consolidated reef matrix, which has rarely been the focus of previous studies. This substrate is the ‘solid’ structure underlying the veneer of living corals. Calcification and accretion of the reef matrix allows reefs to maintain their depth with increases in sea-level [82], furthermore, the consolidated reef matrix is often entirely occupied by algae and other benthic organisms, which are often overlooked using planar methods [38]–[40]. This sampling method also benefits from being relatively straightforward, although video or photographic transects are probably not possible. Unfortunately, benthic transects provide little information on canopy forming species, and as such, are insensitive to the effects of common disturbances [11], [49], [76], [77]. Benthic transects, therefore, provide an accurate picture of reef composition in terms of the area of substratum occupied. Relatively insensitive to changes in canopy forming species, they more accurately portray the role of algae and other benthic organisms on the reef surface.

The chain transects might appear to provide a more balanced approach to benthic sampling as they concurrently assess both the canopy and benthos. In fact, from the perspective of a fish or settling coral planula, which rely on reef surface area for feeding or settlement respectively, it is perhaps the most relevant measure of reef cover. Furthermore, this is the only method, which includes 3-dimensions. However, for some ecological surveys, the double counting of canopies (i.e. including both the upper and lower surfaces) artificially increases the importance of canopy forming corals. Changes in coral cover of the sensitive, canopy-forming species is thus at risk of being over-reported after disturbances. Massive coral colonies, for example, contribute directly to calcification of the reef matrix but due to their low surface area their loss would not be reported to be as important as high surface area, but structurally delicate, canopy forming corals. The main disadvantage with chain transects is, however, logistical. The complexity of the methods means that replication at any site will suffer and less area will be surveyed. Furthermore, the sampling would rely on experienced observers and becomes much more challenging in adverse conditions (a fact to which the authors can attest).

It appears that a combination of survey methods may be most appropriate. For example, stratified sampling using planar and benthic transects together would allow some increased dimensionality with minimal extra effort. Furthermore the problems of the canopy effect could be overcome, as the cover beneath canopies is recorded and therefore can be included as a correction factor if any changes in canopy cover are observed. It must, however, be highlighted that this study is far from comprehensive and a great many more sampling methods currently exist (cf. [4]). Furthermore, new ecological studies will obviously demand the development of new sampling methods. Similarly, sampling in other ecosystems will require different considerations. There is no simple answer to the question, “which sampling method is best?” The most important messages, highlighted by this study are 1) that it is important to match the census technique to the question and, 2) that it is of vital importance to critically evaluate what any method is actually estimating. The greatest danger is following methodological inertia, choosing methods simply because they have been used before.

### Conclusions

The canopy effect has the potential to be pervasive in ecological survey techniques across ecosystems. It is most prevalent in systems dominated by canopy-forming autotrophs as in tropical forests and on coral reefs. The canopy effect, however, is particularly relevant for coral reefs where massive changes are predicted around the world. While planar transects are logistically practical and have a long history of use we must be careful to consider the hidden portions of the benthos, which have the potential to alter our interpretations of the status of ecosystems and how they respond to ever more frequent disturbances. The understories of reefs are not ecologically irrelevant, with diverse algal and coral communities, which, when compared to the canopies, have different susceptibilities and responses to disturbances. Therefore, the current ‘coral-centric’ view of coral reefs, which are often not numerically dominated by corals, might be misleading. A reliance on planar assessments of coral cover as a proxy for reef health should be reassessed. By looking at both planar and benthic cover we can begin to move beyond simple cover estimates to understand the processes that shape benthic configurations and better understand the impacts of the more frequent and severe disturbances that coral reefs are likely to suffer.

### Ethics Statement

All procedures were conducted according to the ethics guidelines of James Cook University, Townsville, and permitting requirements of the Great Barrier Reef Marine Parks Authority, permit number: G10/33755.1.

## Supporting Information

Figure S1 **Results of the three transect types from four reefs around Lizard Island.** **A.** represents Mermaid Cove and **B.** South-Palfrey, two *Acropora-*dominated reefs. **C.** represents Clam Gardens and **D.** Trawler Beach, two *Porites-*dominated reefs. The left hand portion of the graphs shows the algae recorded using the three methods (light grey  =  epilithic algal matrix, dark grey  =  crustose coralline algae). The right hand portion of the graphs shows the results for corals (mid-grey  =  massive and encrusting colonies, black  =  canopy-forming species). Notice the consistent decreases in canopy forming corals and subsequent algal increases (especially EAMs) with the benthic point intercept transect.
(PDF)

Figure S2 **Conceptual model demonstrating the effect of canopy removal on a** ***Porites*** **-dominated reef.** Start points are based on planar transect data. Canopy cover is reduced 10% each generation. End points are based on benthic transect data (with no remaining canopy). The ‘loss’ of canopy cover (difference between planar transects and benthic transects) was almost identical to the apparent increase in benthic algae (6.58% loss vs. 7.23% increase respectively).
(PDF)
