# Supplementary material for: The Roles of Dimensionality, Canopies and Complexity in Ecosystem Monitoring
Source: PLoS One. 2011 Nov 3;6(11):e27307. doi: 10.1371/journal.pone.0027307 (PMC3207849; doi:10.1371/journal.pone.0027307)
Supplement: Figure S1 — Results of the three transect types from four reefs around Lizard Island. A. represents Mermaid Cove and B. South-Palfrey, two Acropora-dominated reefs. C. represents Clam Gardens and D. Trawler Beach, two Porites-dominated reefs. The left hand portion of the graphs shows the algae recorded using the three methods (light grey = epilithic algal matrix, dark grey = crustose coralline algae). The right hand portion of the graphs shows the results for corals (mid-grey = massive and encrusting colonies, black = canopy-forming species). Notice the consistent decreases in canopy forming corals and subsequent algal increases (especially EAMs) with the benthic point intercept transect. (PDF) [file pone.0027307.s001.pdf]

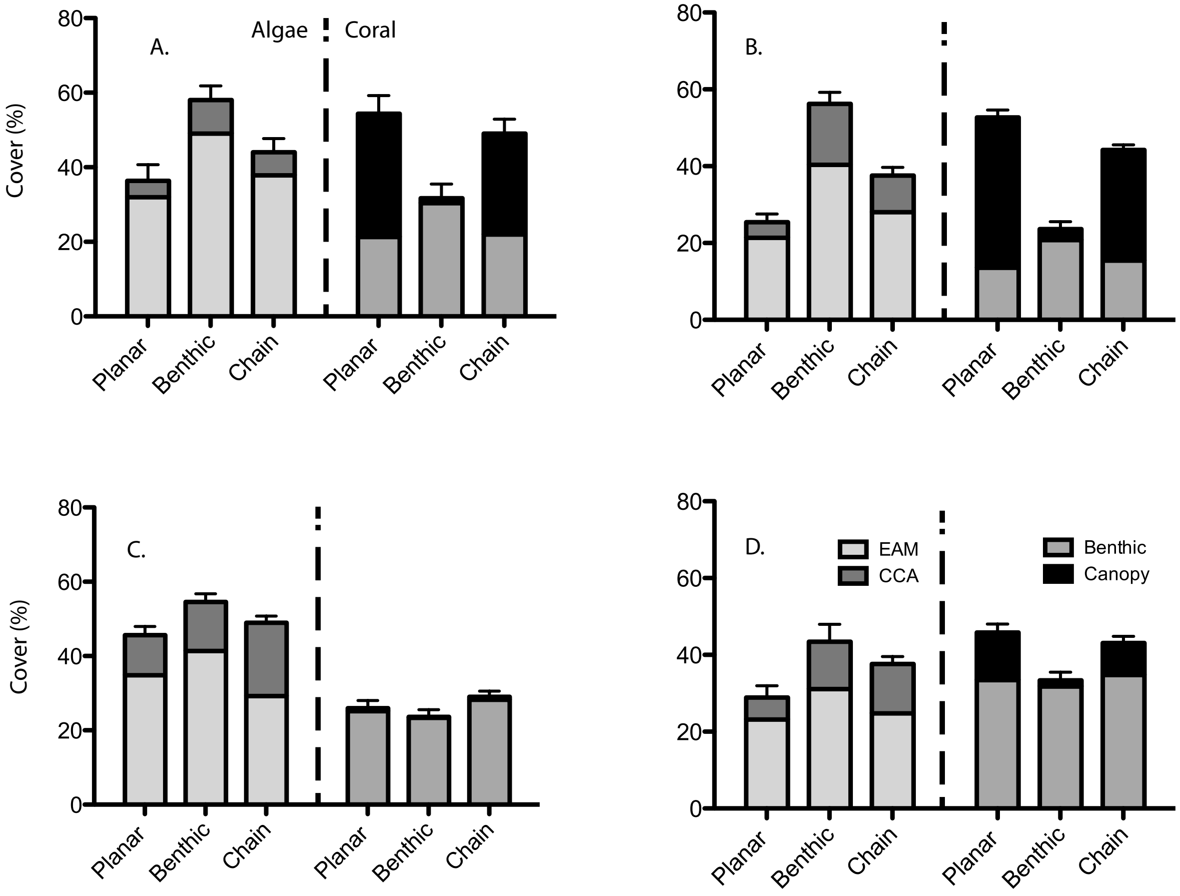

Supplemental material 1: Results of the three transect types from four reefs around Lizard Island on the Northern Great Barrier Reef. **A.** represents Mermaid Cove and **B.** South-Palfrey, two *Acropora* dominated reefs. **C.** represents Clam Gardens and **D.** Trawler Beach, two *Porites* dominated reefs. The left hand portion of the graphs shows the algae recorded using the three methods (light grey = epilithic algal matrix, dark grey = crustose coralline algae). The right hand portion of the graphs shows the results for corals (mid-grey = massive and encrusting colonies, black = canopy-forming species). Notice the consistent decreases in canopy forming corals and subsequent algal increases (especially EAMs) with the benthic point intercept transect.
