# Supplementary material for: The Roles of Dimensionality, Canopies and Complexity in Ecosystem Monitoring
Source: PLoS One. 2011 Nov 3;6(11):e27307. doi: 10.1371/journal.pone.0027307 (PMC3207849; doi:10.1371/journal.pone.0027307)
Supplement: Figure S2 — Conceptual model demonstrating the effect of canopy removal on a Porites -dominated reef. Start points are based on planar transect data. Canopy cover is reduced 10% each generation. End points are based on benthic transect data (with no remaining canopy). The ‘loss’ of canopy cover (difference between planar transects and benthic transects) was almost identical to the apparent increase in benthic algae (6.58% loss vs. 7.23% increase respectively). (PDF) [file pone.0027307.s002.pdf]

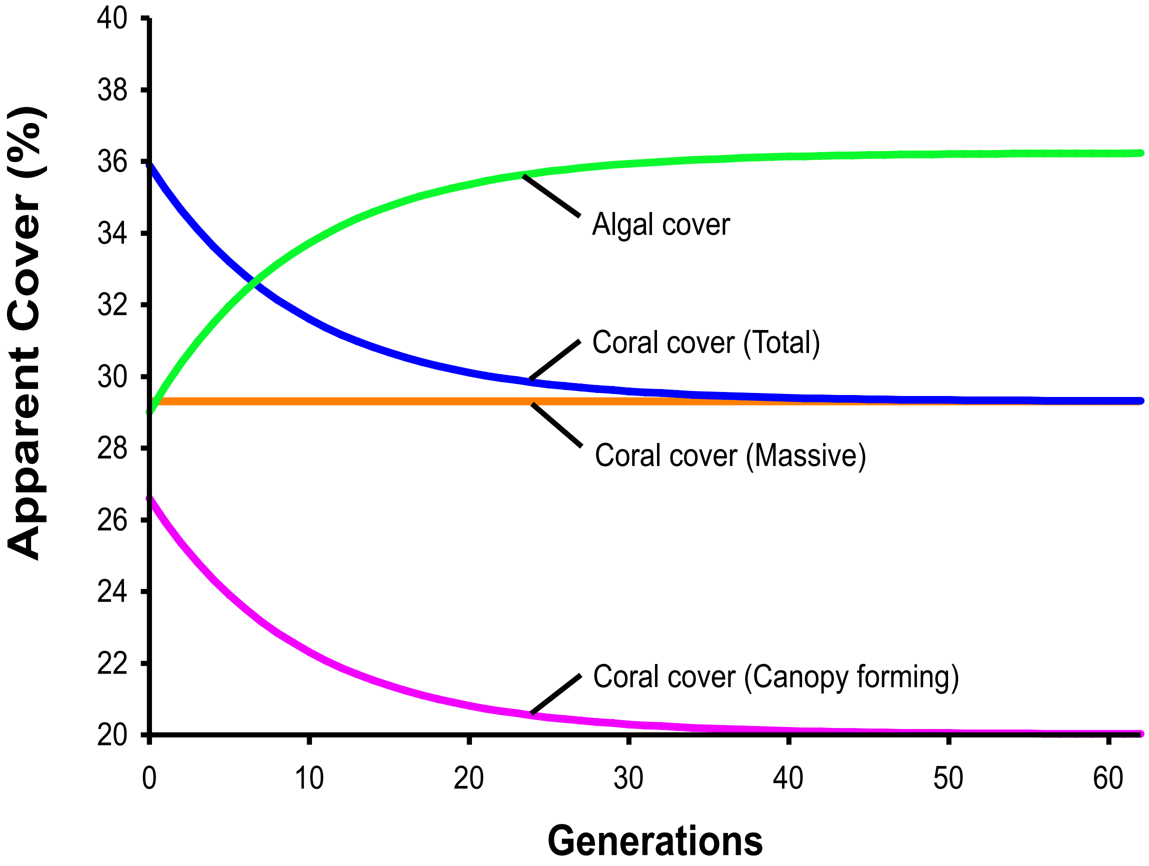

Supplemental material 2: Simple conceptual model demonstrating the effect of canopy removal on planar data collected from a *Porites* dominated reef. Start points are based on planar transect data. Canopy cover is reduced 10% each generation. End points are based on benthic transect data (with no remaining canopy). The 'loss' of canopy cover (difference between planar transects and benthic transects) was almost identical to the apparent increase in benthic algae (6.58% loss vs. 7.23 increase respectively).
